# Factors associated with reproductive health care utilization among Ghanaian women

**DOI:** 10.1186/1472-698X-12-29

**Published:** 2012-11-07

**Authors:** David Doku, Subas Neupane, Paul Narh Doku

**Affiliations:** 1 Department of Population and Health, University of Cape Coast, Private Mail Bag, University Post Office, Cape Coast, Ghana; 2 School of Health Sciences, FI – 33014 University of Tampere, Tampere, Finland; 3 Department of Psychology, Regent University of Science and Technology, Accra, Ghana

**Keywords:** Antenatal care, Maternal health, Timing of antenatal care visit, Type of delivery assistance

## Abstract

**Background:**

This study investigates factors determining the timing of antenatal care (ANC) visit and the type of delivery assistant present during delivery among a national representative sample of Ghanaian women.

**Method:**

Data for the study was drawn from the women questionnaire (N=4,916) of the 2008 Ghana Demographic and Health Survey among 15–49-years-old women. Multivariate logistic regression analysis was used to explore factors determining the type of delivery assistance and timing of ANC visit for live births within five years prior to the survey.

**Results:**

Majority of Ghanaian women attended ANC visit (96.5%) but many (42.7%) did so late (after the first trimester), while 36.5% had delivery without the assistance of a trained personnel (30.6%) or anyone (5.9%). Age (OR=1.5, CI=1.1-1.9, OR for 25-34-year-olds compared to 15-24-year-olds), religion (OR=1.8, CI=1.2-2.8, OR for Christians versus Traditional believers) wealth index (OR=2.6, CI=1.7-3.8, OR for the richest compared to the poorest) were independently associated with early ANC visit. Likewise, age, place of residence, education and partner’s education were associated with having a delivery assisted by a trained assistant. Also, Christians (OR=1.8, CI=1.1-3.0) and Moslems (OR=1.9, CI=1.1-3.3) were more likely to have trained delivery assistants compared to their counterparts who practised traditional belief. Furthermore, the richer a woman the more likely that she would have delivery assisted by a trained personnel (OR=8.2, CI= 4.2-16.0, OR for the richest in comparison to the poorest).

**Conclusions:**

Despite the relatively high antenatal care utilisation among Ghanaian women, significant variations exist across the socio-demographic spectrum. Furthermore, a large number of women failed to meet the WHO recommendation to attend antenatal care within the first trimester of pregnancy. These findings have important implications for reducing maternal mortality ratio by three-quarters by the year 2015.

## Background

Research has demonstrated that antenatal care (ANC) is beneficial to women [1,2]. In addition to vaccination for pregnant women to immunise them against some of the childhood killer diseases, during antenatal care women are given advice regarding the changes that they will go through and how their lifestyles may affect the health of the developing foetus. Antenatal care also provides encouragement and support for women, and as well, screening programmes that are incorporated into such care may lead to the detection of problems that may arise during the pregnancy thereby reducing complications or preparing for them in high risk groups [3,4]. Studies have shown that women who use ANC services have higher likelihood of having childbirth in health facility or giving birth in the presence of trained birth attendants compared to those who do not use ANC services [5,6].

The fifth Millennium Development Goal (MDG) of United Nations (UN) is to improve maternal health and this is to be achieved by reducing maternal mortality ratio by three-quarters between 1990 and 2015 [7]. Achieving good maternal health requires quality reproductive health services and a series of well-timed interventions. In most developing countries, the timing of ANC is very essential because it offers an opportunity for the prevention of mother to child HIV/AIDS transmission.

On the other hand, preventing maternal mortality and morbidity to ensure that women receive appropriate care during delivery still remained one of the critical issues. All deliveries should be assisted by personnel with the relevant skills to handle typical deliveries safely and to recognize the onset of complications [8]. Skilled personnel should either provide the necessary treatment, if within their capacities, or refer the woman to emergency care if treatment is beyond their capacities [9]. One of the reports of United Nations Children's Fund (UNICEF) shows that, 4 in 10 of all births worldwide are not assisted by skilled health personnel [9]. Sub-Saharan Africa and South Asia have the lowest levels of skilled birth assistants and bear the greatest burden of maternal mortality [9]. In contrast, 99 per cent of deliveries in developed countries are assisted by skilled personnel.

Ghana’s maternal mortality ratio (MMR) of 300–549 per 100 000 live births [10] is comparably high despite efforts being made to meet the MDG 5. A major challenge has been to increase skilled assistants for home deliveries assisted by unskilled traditional birth personnel, a practice that is still the preferred mode of delivery for majority of pregnant women, especially in rural areas.

Previous studies have shown fairly consistent relationship between socio- demographic factors (such as maternal age, parity, maternal educational attainment, place of residence and ethnicity) and institutional delivery as well as early ANC use [11-14]. Many studies from Ghana have attempted to analyse causes of maternal mortality with respect to socio-demographic characteristics [15-18]. However, detailed studies from Ghana as well as African countries examining determinants of maternal health care in general and ANC in particular are scarce.

This study seeks to use a national representative data from the Ghana Demographic Health Survey (GDHS) to investigate factors determining the timing of antenatal care visit and the type of delivery assistance among Ghanaian women. The findings of this study would enlighten our understanding of ANC utilisation and would contribute to the promotion of maternal health among women.

## Methods

Data for this study came from the 2008 Ghana Demographic and Health Survey (GDHS), which is a nationwide representative sample of women aged 15-49-year-old. The survey used a two stage sample based on the 2000 Population and Housing Census to produce separate estimates for key indicators for each of the ten regions in Ghana. Over 12000 households were selected for the survey. Each household selected for the GDHS was eligible for interview with the household questionnaire, and a total of 11,778 households were interviewed. In half of the households selected for the survey, all eligible women aged 15-49-year-old were interviewed with the women’s questionnaire. A total of 4,916 women aged 15-49-year-old were interviewed. The data collection took place over a three-month period, from early September to late November, 2008. The present study used the women questionnaire (N=4,916). The analysis was restricted to the most recent birth to 15-49-years-old women who gave live births within the five years preceding the survey (N=2099). The study protocol was approved by the Ghana Health Service Ethical Review Committee in Accra, Ghana. Details of the GDHS are published elsewhere [19].

### Study variables

Two dichotomous dependent variables were used

a) Timing of ANC visit, derived from the following questions: “How many months pregnant were you when you first received antenatal care for this pregnancy?” Timing of ANC visit was recoded as “Late” when the visit took place during the second or third trimester, and “Early” when it occurred during the first trimester. The World Health Organization (WHO) recommends that for the majority of normal pregnancies, ANC should consist of at least four visits during the course of the pregnancy, the first of which should occur within the first trimester [20].

b) Type of delivery assistance derived from the questions; “Who assisted with the delivery of (NAME OF CHILD)?” Type of delivery assistance was recoded into three categories: “None” for no assistance and assistance from relatives or others without professional skills; “TBA” for assistance from traditional birth assistants (TBA); and “Skilled professional” for assistance from a doctor, nurse, midwife, auxiliary midwife or community health officer. For the logistic regression analyses two categories were made: delivery assisted by “trained personnel” and delivery assisted by “untrained personnel” (no care and untrained personnel).

With reference to previous studies [21,22], the independent variables used in this study include number of ANC visits, education (coded as none, primary and secondary/higher), household wealth, represented by wealth index (in five categories from poorest to richest), urban–rural residence, marital status, parity, age and partner’s education (coded as none, primary and secondary/higher). The wealth index was constructed based no previous studies, e.g. Filmer and Pritchett [23]. Although no validity study of the wealth index has been conducted for this particular index, Filmer and Pritchett [23] have shown that such measures are valid and reliable.

### Statistical analysis

First, Pearson’s Chi-square test was used to study the relationship between socio-demographic factors and the early antenatal care and delivery assisted by trained personnel. Second, logistic regression was used to study the associations between socio-demographic variables and the two outcome variables. Bivariate logistic analyses were computed to investigate the associations between socio-demographic factors and early antenatal care and delivery assisted by trained personnel (Model 1). Next, adjusted models were computed as follows: all the socio-demographic variables that were statistically significant at the bivariate level (Model 1) were simultaneously adjusted in relation to the two outcome variables (Model 2). Regarding the outcome variable delivery assisted by trained personnel, in addition to the background variables in Model 2, the timing of ANC visit was adjusted for (Model 3), and a final multivariate logistic regression model consisting of Model 3 plus partner’s education was computed. For the outcome variable early antenatal care, its final multivariate logistic regression model (Model 3) consisted of Model 2 plus partner’s education. The results of the Logistic regression analyses are presented as odds ratios (OR) with 95% confidence intervals (CI). Statistical significance was defined as a two-sided P-value <0.05 in all analyses. All analyses were weighted. The Statistical Programme for Social Sciences (SPSS) software version 16 was used for the statistical analyses.

## Results

The mean age of respondents in this study was 30.13 year. Forty-three per cent (43%) of women attended antenatal care late (after the first trimester of pregnancy). On the average, the timing of the first antenatal care visit occurred after the first trimester of pregnancy (3.8 months) and the mean number of visits during pregnancy was 7.8 times.

Of all women who gave birth within the 5 years preceding the survey, 36.5% of them were not assisted by trained personnel during the last birth. All the background variables in this study, with the exception of marital status, were related to both the type of delivery assistance (trained vs untrained) and the timing of ANC visit (early vs late), Table 1.

**Table 1:** Distribution of socio-demographic factors in relation to the timing of antenatal care visit and type of delivery assistance among Ghanaian women

**Socio-demographic factors**	**Number**	**Percentage**	**Delivery Assistance**		**Timing of antenatal care**	
			**Trained**	**Untrained**	**Early**	**Late**
**Age (N=2099)**			P=0.004		P<0.001	
15-24 years	505	24.0	22.7	26.4	21.3	27.5
25-34 years	982	46.8	49.6	42.0	50.9	41.9
35-49 years	612	29.1	27.7	31.6	27.8	30.6
**Place of residence (N=2099)**			P<0.001		P<0.001	
Urban	844	40.2	54.4	15.7	44.8	36.0
Rural	1255	59.8	45.6	84.3	55.2	64.0
**Parity (N=2098)**			P<0.001		P<0.001	
1	467	22.3	25.9	16.1	23.8	21.3
2	436	20.8	22.7	17.4	23.9	16.7
3	349	16.6	17.0	16.1	17.2	15.9
4 or more	846	40.3	34.4	59.5	35.8	46.0
**Marital status (N=2099)**			P=0.166		P=0.976	
Never married	129	6.1	6.8	4.8	6.2	6.1
Currently married	1837	87.5	86.6	89.0	87.8	87.7
Formerly married	133	6.3	6.5	6.1	6.1	6.3
**Education (N=2097)**			P<0.001		P<0.001	
No education	647	30.8	20.3	49.0	25.2	37.1
Primary	511	24.1	22.8	27.2	23.8	24.1
Secondary or higher	939	44.8	56.9	23.8	51.0	38.8
**Religion (N=2098)**			P<0.001		P<0.001	
Other	90	4.3	3.5	5.8	3.6	4.3
Moslem	378	18.0	16.3	21.0	15.2	22.7
Traditional	126	6.0	2.3	12.4	3.7	8.1
Christian	1504	71.7	78.0	60.8	77.5	64.8
**Wealth Index (N=2099)**			P<0.001		P<0.001	
Poorest	480	22.9	10.3	44.6	17.3	28.5
Poorest	461	22.0	19.2	26.5	19.9	24.0
Middle	400	19.1	20.3	17.0	19.1	19.6
Rich	436	20.8	27.2	9.8	23.1	19.0
Richest	322	15.3	22.9	2.1	20.6	9.0
**Partner’s education (N=1878)**			P<0.001		P<0.001	
No education	483	25.7	15.0	44.3	19.8	31.8
Primary	159	8.5	7.1	10.8	7.1	10.0
Secondary or higher	1236	65.8	77.9	44.9	73.0	58.2
**Timing of antenatal care visit (N=2014)**			P<0.001			
Early (within first trimester)	1154	57.3	37.8	52.0		
Late	860	42.7	62.2	48.9		
**Number of antenatal visits (N=2053)**			P<0.001		P<0.001	
No visit	72	3.5	0.9	8.2		
1-3 visits	338	16.5	9.8	28.2	6.5	31.1
4-6 visits	900	43.9	41.2	48.3	41.4	51.2
6 visits or more	740	36.1	48.0	15.3	52.0	17.7
**Type of delivery assistance (N=2096)**					P<0.001	
Trained assistants	1330	63.5			70.9	57.8
Traditional birth assistants	642	30.6			24	35.6
No one	124	5.9			4.4	6.5

### Factors associated with early antenatal care visit

The following socio-demographic factors were associated with early ANC visit bivariately; age, place of residence, parity (borderline significance), education, religion, wealth index and partner’s education (Table 2, Model 1). In multivariate analyses after controlling for each of these socio-demographic factors that were statistically significant at the bivariate level (Table 2, Model 2), age (OR=1.5, CI=1.2-1.9, OR for 25–34 year olds compared to 15–24 year olds), religion (OR=1.8, CI=1.2-2.7, OR for Christians versus Traditional believers) and wealth index (OR=3.4, CI=2.2-5.2, OR for the richest compared to the poorest) increased the likelihood of having early ANC visit. When partner’s education was controlled for those factors that independently predicted early ANC, the following were found: age (OR=1.5, CI=1.1-1.9, OR for 25–34 year olds compared to 15–24 year olds), religion (OR=1.8, CI=1.2-2.8), OR for Christians versus Traditional believers) wealth index (OR=2.6, CI=1.7-3.8), OR for the richest compared to the poorest) were independently associated with early ANC visit. A woman’s partners’ education was also independently associated with early ANC visit.

**Table 2:** Odds ratios (OR) and 95% confidence intervals (CI) for factors associated with early antenatal care visit among Ghanaian women in 2008

**Background variables**	**Model 1**	**Model 2**	**Model 3**
	**OR (95% CI)**	**OR (95% CI)**	**OR (95% CI)**
**Age**			
15-24 years	1.0	1.0	1.0
25-34 years	1.6 (1.2-1.9)	1.5 (1.2-1.9)	1.5 (1.1-1.9)
35-49 years	1.2 (0.9-1.5)	1.2 (1.0-1.6)	0.2 (0.9-1.6)
**Place of residence**			
Rural	1.0	1.0	
Urban	1.4 (1.2-1.7)	1.2 (1.0-1.5)	
**Parity**			
1	1.0		
2	1.3 (0.9-1.7)		
3	1.0 (0.7-1.3)		
4 or more	0.7 (0.5-0.9)		
**Marital status**			
Never married	1.0		
Currently married	1.0 (0.7-1.4)		
Formerly married	1.0 (0.6-1.6)		
**Education**			
No education	1.0	1.0	
Primary	1.4 (1.1-1.8)	1.2 (1.0-1.6)	
Secondary or Higher	1.9 (1.5-2.3)	1.2 (1.0-1.6)	
**Religion**			
Traditional	1.0	1.0	1.0
Moslem	1.5 (1.0-2.3)	1.2 (0.8-1.9)	1.4 (0.8-2.2)
Christian	2.6 (1.8-3.9)	1.8 (1.2-2.7)	1.8 (1.1-2.8)
**Wealth Index**			
Poorest	1.0	1.0	1.0
Poorer	1.4 (1.0-1.8)	1.2 (0.9-1.6)	1.0 (0.7-1.4)
Middle	1.6 (1.2-2.1)	1.4 (1.0-2.0)	1.2 (0.8-1.6)
Richer	2.0 (1.5-2.6)	1.8 (1.2-2.6)	1.6 (1.1-2.2)
Richest	3.7 (2.7-5.2)	3.4 (2.2-5.2)	2.6 (1.7-3.8)
**Partner’s education**			
No education	1.0		1.0
Primary	1.1 (0.8-1.7)		1.0 (0.6-1.5)
Secondary or Higher	2.0 (1.6-2.5)		1.3 (1.1-1.8)

Model 1=bivariate analyses.

Model 2 =Multivariate analyses of all independent variable statistically. significant at the bivariate level.

Model 3=Model 2 plus partner’s education.

### Factors associated with trained delivery assistant

The socio-demographic variables age, place of residence, religion and wealth index all were statistically significantly associated with having trained delivery assistant during the most recent birth bivariately (Table 3, Model 1) and multivariately after controlling for the effects of the other socio-demographic variables that statistically significantly predicted having trained delivery assistant available during delivery (Table 3, Model 2), the other socio-demographic variables that statistically significantly predicted having trained delivery assistant plus the timing of first ANC visit (Table 3, Model 3), and the other socio-demographic variables that statistically significantly predicted having trained delivery in addition to the timing of the first ANC visit plus partner’s education. Women aged 25-34-years-old were more likely to have trained delivery assistant compared to 15-24-years-old women but no statistically significant difference was found between the oldest women (35–49 years) and the younger ones (15-24-years-old). Urban residency increased the probability of having a delivery assisted by a trained assistant compared to rural residency (OR=2.1, CI= 1.5-2.9). Christians (OR=1.8, CI=1.1-3.0) and Moslems (OR=1.9, CI=1.1-3.3) women were more likely to have trained delivery assistants compared to their counterparts who practised traditional belief. Furthermore, the richer a woman the more likely that she would have delivery assisted by a trained assistant (OR=8.2, 4.2-16.0, OR for the richest in comparison to the poorest).

**Table 3:** Odds ratios (OR) and 95% confidence intervals (CI) for factors associated with delivery assisted by trained personnel among Ghanaian women in 2008

**Background variables**	**Model 1**	**Model 2**	**Model 3**	**Model 4**
	**OR (95% CI)**	**OR (95% CI)**	**OR (95% CI)**	**OR (95% CI)**
**Age**				
15-24 years	1.0	1.0	1.0	1.0
25-34 years	1.4 (1.1-1.7)	1.8 (1.3-2.4)	1.6 (1.1-2.2)	1.6 (1.2-2.3)
35-49 years	1.0 (0.8-1.3)	2.2 (1.4-3.2)	2.0 (1.3-3.0)	2.0 (1.3-3.1)
**Place of residence**				
Rural	1.0	1.0	1.0	1.0
Urban	6.4 (5.1-8.0)	2.2 (1.6-3.0)	2.3 (1.7-3.2)	2.1 (1.5-2.9)
**Parity**				
1	1.0	1.0	1.0	1.0
2	0.6 (0.5-0.9)	0.8 (0.5-1.1)	0.8 (0.5-1.1)	0.8 (0.5-1.2)
3	0.4 (0.3-0.6)	0.7 (0.5-0.9)	0.7 (0.5-1.0)	0.7 (0.5-1.1)
4 or more	0.2 (0.1-0.3)	0.5 (0.3-0.7)	0.5 (0.3-0.8)	0.6 (0.4-0.9)
**Marital status**				
Never married	1.0			
Currently married	0.7 (0.5-1.0)			
Formerly married	0.7 (0.4-1.2)			
**Education**				
No education	1.0	1.0	1.0	1.0
Primary	2.0 (1.6-2.5)	1.2 (0.9-1.6)	1.2 (0.9-1.7)	1.0 (0.8-1.4)
Secondary or Higher	5.8 (4.6-7.2)	2.1 (1.6-2.8)	2.1 (1.5-2.8)	1.8 (1.3-2.5)
**Religion**				
Traditional	1.0	1.0	1.0	1.0
Moslem	4.3 (2.7-6.8)	2.1 (1.3-3.6)	1.8 (1.1-3.2)	1.9 (1.1-3,3)
Christian	7.1 (4.7-10.9)	2.4 (1.4-3.8)	2.2 (1.3-3.6)	1.8 (1.1-3.0)
**Wealth Index**				
Poorest	1.0	1.0	1.0	1.0
Poorer	3.1 (2.4-4.1)	2.2 (1.7-3.1)	2.3 (1.7-3.1)	2.1 (1.5-3.0)
Middle	5.1 (3.8-6.8)	2.4 (1.7-3.4)	2.3 (1.6-3.3)	2.0 (1.4-3.0)
Richer	12.0 (8.7-16.5)	4.5 (3.0-6.7)	3.9 (2.6-5.9)	3.5 (2.2-5.4)
Richest	46.2 (27.1-78.9)	11.1 (6.0-20.7)	5.3 (1.7-3.2)	8.2 (4.2-16.0)
**Timing of antenatal care visits**				
Late	1.0		1.0	
Early	1.8 (1.5-2.1)		0.8 (0.7-1.0)	
**Partner’s education**				
No education	1.0			1.0
Primary	2.0 (1.4-2.8)			1.6 (1.1-2.5)
Secondary or Higher	5.1 (4.1-6.4)			1.7 (1.2-2.3)

Model 1=bivariate analyses.

Model 2 =Multivariate analyses of all independent variable statistically significant at the bivariate level.

Model 3= Model 2 plus timing of antenatal care visits.

Model 4=Model 3 plus partner’s education.

At bivariate level, having higher parity decreased the chances of having trained delivery assistant compared to when parity is one. In the multivariate model after controlling for the effects of the other socio-demographic variables that statistically significantly predicted having trained delivery in addition to the timing of the first ANC visit plus partner’s education, only the difference for those who had more than four children was retained (OR=0.5, CI=0.3-0.8). Similarly, women with primary education (OR=2.0, CI=1.6-2.5) and secondary education or higher (OR=5.8, CI=4.6-7.2) had higher probability of having a trained delivery assistant compared to those without any formal education. However, at the multivariate level, there was only statistically significant difference between those who had secondary education or higher (OR= 1.8, CI=1.3-2.5) compared to those with no formal education (Table 3, Model 4).

## Discussion

A high proportion of Ghanaian women used antenatal care but many started after the first trimester of pregnancy. Also, a considerable proportion of women were not assisted by trained personnel during delivery and more than two-thirds of women either did not attend antenatal care at all or attended late. Age, religion, wealth index and partner’s education were all associated with early ANC visit. This study found that several socio-demographic factors were associated with the probability of a woman having a trained assistant during delivery. Age, education, wealth, religion and partner’s education were independently associated with delivery assisted by a trained assistant. The older a woman, the more likely that she would have a delivery assisted by trained assistant. No consistent trend was found between parity and delivery assisted by trained personnel. Women of the Christian and Islamic faiths were both more likely to have delivery assisted by trained personnel compared to Traditional faith practitioners. The richer a women, the higher the probability of having a delivery assisted by trained personnel. Furthermore, women whose partners had primary or secondary education and higher had higher chances of having delivery assisted by trained personnel compared to those whose partners had no education.

In this study, women aged 25-34-years-old had higher probability of having early ANC visit compared to those aged 15-24-years-old. Similarly, the older a woman, the more likelihood it was that she would have a delivery assisted by a trained personnel. Maternal age has been shown to affect both the timing of ANC visit and ANC care utilisation in general [22-25]. However, there is no consistency in the findings. While some studies found that younger age increases the chances of early antenatal care, delivery assisted by trained personnel and high utilization of antenatal care services [24], others found otherwise [26], and yet others found no variation in maternal care utilisation by age [25,27]. Plausibly, the socio-cultural context of both age and maternal health care utilisation may explain the differences in the findings.

Consistent with previous studies in Nepal [24], Kenya [28] and elsewhere [21], urban residency was found to increase the likelihood of both early ANC visit as well as delivery assisted by trained personnel. Disparities in the distribution of health care services in most developing countries to the disadvantage of those in rural settings and differences in the exposure to health information to the merits of urban women could account for the rural–urban differences in antenatal care utilisation.

A woman’s secondary or tertiary education places her at a greater chance of having trained personnel at delivery compared to having no education. In a review article on factors affecting antenatal care utilisation in developing countries, Simkhada, Teijlingen, Porter, et al., [22] identified maternal education as an important factor affecting the uptake of antenatal care among women in developing countries. Similarly, in a recent study among Nepalese women, Neupane and Doku [24] reported that women with no education were more likely to have no maternal service use at all compared to those with primary education or more. Lack of knowledge about the importance of maternal and child health has been cited as hindering women from using antenatal services [21]. Interestingly, the association between antenatal care utilisation and education was not only related to women’s education but also their partners’ education, such that the higher the partner’s education the higher the likelihood that a woman would have an early antenatal care or have a delivery assisted by trained personnel, independent of the woman’s education. This accords with previous findings from various developing countries [22] and highlights the importance of male partners in promoting maternal health.

Christianity favoured both early antenatal care and delivery assisted by trained personnel compared to Traditional faith practice. Also, Moslems were more likely to have delivery assisted by trained personnel compared with Traditional faith practitioners. In a recent study among Nepalese women [24], it was found that religion (Hinduism) was associated with early antenatal care visits and high number of prenatal care visits, confirming the findings in the present study regarding differences in reproductive health utilisation by religious beliefs. Religion may act as a network where social values are shared among its members. Hence this could explain the differences in antenatal care utilisation by religion. This finding has important implications for health promotion among religious groups, especially in African countries where people are strongly attached to faith groups. Being an organised entity, it may be relatively easier to channel health promotion messages through their well respected leaders (imams and pastors) to the congregations.

A woman’s household socioeconomic status, measured by wealth index, was associated with the timing of ANC visit and the type of delivery assistance. Women from higher wealth index household were more likely to start antenatal care in the first trimester or have delivery attended to by trained personnel compared to those from lower wealth index households. Other previous studies have found similar results [22,24,29]. The mechanism underlying the relationship between wealth index and antenatal care utilisation could be explained by affordability along with higher chances of health information among women of higher SES compared to their lower SES counterparts. Listwise deletion was employed in the handling of missing data. This leads to bias, especially where there are large missing data. However, as missing data was low in this study (<1% for most variables), the listwise deletion of missing data is unlikely to affect the estimates or alter the conclusions reached. A further limitation of this study is that women who died after giving birth were not captured in the survey. Although the effect of this is unknown, it is likely to underestimate the SES differences found.

## Conclusions

Although majority of Ghanaian women attended antenatal care, many of them (nearly one out of every two) failed to meet the WHO recommendation that women should seek antenatal care during the first trimester of pregnancy. This implies that many women are missing the opportunity that antenatal care offers in detecting pregnancy related problems early. This study revealed that age, religion, wealth index and a woman’s partner’s education were associated with early ANC visit. Furthermore, age, place of residence, religion, education, wealth index and partner’s education were associated with having delivery attended to by trained personnel. These findings should be taken into consideration when designing programmes aimed at promoting antenatal care utilisation across the socio-demographic spectrum. These findings also have important policy implications for meeting the MDG number 5 of improving maternal health by reducing maternal mortality ratio by three-quarters by 2015. Health education programmes should be undertaken to improve women’s awareness of ANC. The Ministry of Health should provide mobile ANC services similar to the mobile postnatal “weighing services” that are provided in communities. This will enable remote communities to access the services. The training of traditional birth attendants is also recommended.

## Competing interests

All authors declare that they have no competing interest.

## Authors’ contributions

DD and SN were involved in the conception of the study. DD conducted the statistical analysis. DD and SN drafted the manuscript. DD, PND and SN were involved in the interpretation of data and the critical revision of the manuscript for important intellectual content. All authors gave final approval of the version to be published.

## Pre-publication history

The pre-publication history for this paper can be accessed here:

http://www.biomedcentral.com/1472-698X/12/29/prepub
